# Comparing the Diagnostic Accuracy of Two Cognitive Screening Instruments in Different Dementia Subtypes and Clinical Depression

**DOI:** 10.3390/diagnostics9030093

**Published:** 2019-08-08

**Authors:** Rónán O’Caoimh, D. William Molloy

**Affiliations:** 1 Centre for Gerontology and Rehabilitation, University College Cork, St Finbarr’s Hospital, Douglas road, T12 XH60 Cork City, Ireland; 2 Department of Geriatric Medicine, Mercy University Hospital, Grenville Place, T12 WE28 Cork City, Ireland

**Keywords:** dementia, mild cognitive impairment, screening, accuracy, standardised mini-mental state examination, quick mild cognitive impairment screen

## Abstract

Short but accurate cognitive screening instruments are required in busy clinical practice. Although widely-used, the diagnostic accuracy of the standardised Mini-Mental State Examination (SMMSE) in different dementia subtypes remains poorly characterised. We compared the SMMSE to the Quick Mild Cognitive Impairment (Q*mci*) screen in patients (*n* = 3020) pooled from three memory clinic databases in Canada including those with mild cognitive impairment (MCI) and Alzheimer’s, vascular, mixed, frontotemporal, Lewy Body and Parkinson’s dementia, with and without co-morbid depression. Caregivers (*n* = 875) without cognitive symptoms were included as normal controls. The median age of patients was 77 (Interquartile = ±9) years. Both instruments accurately differentiated cognitive impairment (MCI or dementia) from controls. The SMMSE most accurately differentiated Alzheimer’s (AUC 0.94) and Lewy Body dementia (AUC 0.94) and least accurately identified MCI (AUC 0.73), vascular (AUC 0.74), and Parkinson’s dementia (AUC 0.81). The Q*mci* had statistically similar or greater accuracy in distinguishing all dementia subtypes but particularly MCI (AUC 0.85). Co-morbid depression affected accuracy in those with MCI. The SMMSE and Q*mci* have good-excellent accuracy in established dementia. The SMMSE is less suitable in MCI, vascular and Parkinson’s dementia, where alternatives including the Q*mci* screen may be used. The influence of co-morbid depression on scores merits further investigation.

## 1. Introduction

Although short cognitive screening instruments (CSIs) such as the Mini-Mental State Examination (MMSE) [1] and its standardised version, the SMMSE [2,3] are widely used in clinical practice and research studies, their accuracy and hence suitability for use in detecting different dementia subtypes is poorly characterised [4]. Numerous studies including recent systematic reviews show that the MMSE has poor accuracy and that an alternative instrument should be used to identify those with mild cognitive impairment (MCI) [5], a prodromal state characterised by cognitive deficits without loss of social or occupation function and before the onset of dementia [6]. Other instruments such as the Memory Alteration Test [7], the Quick Mild Cognitive Impairment (Q*mci*) screen [8] and Montreal Cognitive Assessment (MoCA) [9] are recommended as alternatives [5,10]. The MMSE has a “floor” effect such that a score of zero does not always support dementia and a “low ceiling” effect such that a normal score does not always mean normal cognition [11]. It is also influenced by age and education meaning that it is poorly sensitive when used among older and less educated adults [12,13]. It also has poor reliability, which led to the development of the SMMSE. Despite these issues, the MMSE remains the most widely used CSI in clinical practice [14].

Given that the MMSE and SMMSE cover a limited number of cognitive domains [2,3], their diagnostic accuracy in different types of dementia is unclear. The SMMSE lacks a subtest measuring executive function, which suggests that it is less useful in those with Parkinson’s disease cognitive impairment [15] and is overly weighted towards language skills, making it less useful in frontotemporal dementia [16]. Compared with other short CSIs such as the MoCA, the MMSE has poor accuracy in detecting vascular cognitive impairment, which is often characterised by impaired attention, executive and visuospatial dysfunction, all poorly assessed with the MMSE subtests [17]. Similarly, the pentagon task may lack accuracy in post-stroke, frontotemporal and subcortical dementias [18]. 

Low mood and depression may also impact on cognition and may precede onset of cognitive decline, affecting executive function, memory, and attention [19,20]. While evidence suggests that depression in MCI is associated with disease progression, the effects of depression on cognitive screening are poorly characterised [21]. Limited data suggests that mood may negatively influence MMSE scores, underestimating true performance [22]. The extent to which depression affects the results of the MMSE and other CSIs and their subtests is however, poorly understood and the extent to which different dementia subtype scores are influenced by the presence of comorbid depression is not known. 

Although several studies have compared the diagnostic accuracy of the newer and shorter Q*mci* screen and shown it is better able to differentiate MCI from mild dementia and normal cognition than the SMMSE [8,13], to date, this has only been examined in those with Alzheimer’s and vascular type dementia. The objective of this study is therefore to explore the performance (diagnostic accuracy) of these instruments across a broad range of different dementia subtypes, and in patients with MCI and those with and without depression. More broadly, this also serves to investigate the extent to which CSI’s perform differently in these settings. 

## 2. Methods 

### 2.1. Data Collection

This study compared the SMMSE and the Q*mci* screen in patients (*n* = 3020) obtained from three geriatric and memory clinics in Canada over the decade between 1999 and 2010. Data were collected and analysed retrospectively from two clinic databases and a randomised controlled clinical trial dataset: The Geriatric Assessment Tool (GAT) [23], the Q*mci* screen original validation [8], and the Doxycycline and Rifampicin for Alzheimer’s Disease (DARAD) Trial databases [24]. Recruitment processes from all three studies have been published previously [8,23,24]. In summary, the GAT is a customised software application that automates clinicians’ outpatient reviews [23]. These data were collected in outpatient geriatric and memory clinics in two university hospitals in Ontario, Canada between 1999 and 2010. It contains approximately 8000 individual assessments from 1749 people aged 41–104 years. The Q*mci* screen validation database includes patients referred for assessment of cognition aged ≥55 years and recruited from four memory clinics in Ontario Canada [8]. The DARAD was a multi-centre, blinded, randomised trial conducted between 2006 and 2010, comparing the effect of rifampicin and doxycycline to placebo on the progression of AD [24]. The DARAD database includes patients ≥50 years with mild to moderate AD recruited from 14 centres across Canada. All three studies were led by the same principle investigator (D.W.M) and each participant underwent similar comprehensive work-up including laboratory investigations, neuropsychological assessment and neuroimaging where appropriate [23,24]. Ethical approval was obtained in advance for all three studies and participants provided informed written consent. 

### 2.2. Participants

**Figure 1:**
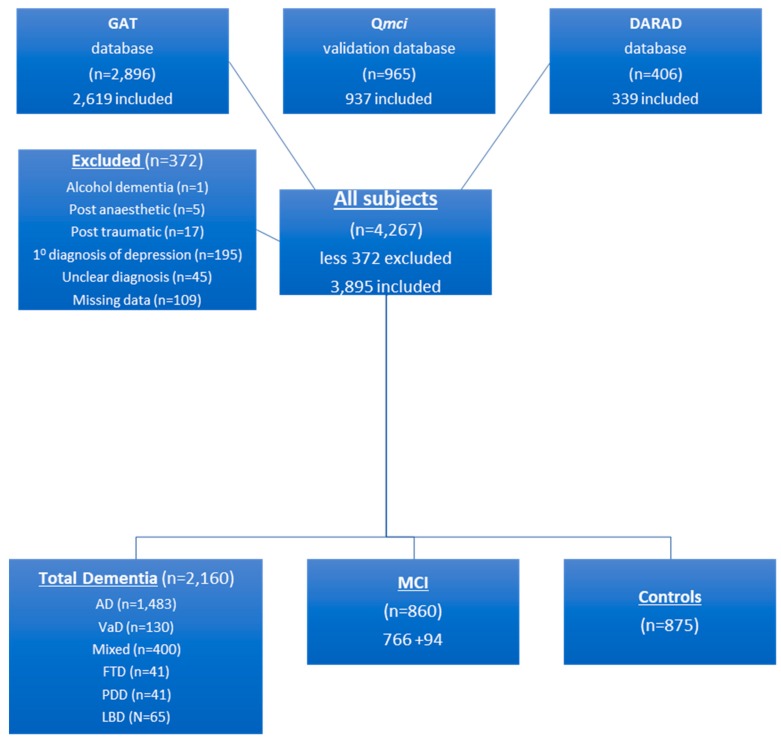
Flow chart presenting the recruitment of participants pooled from three data sets: The Geriatric Assessment Tool (GAT), Quick Mild Cognitive Impairment (Q*mci*) Screen and Doxycycline and Rifampicin for Alzheimer’s Disease (DARAD) trial databases; it includes the number of controls, those with mild cognitive impairment (MCI) and specific dementia subtypes: Alzheimer’s disease (AD), vascular dementia (VaD), mixed dementia, frontotemporal dementia (FTD), Parkinson’s disease dementia (PDD) and Lewy body dementia (LBD) subtypes.

Participants were included in this analysis if both their SMMSE and Q*mci* screen scores were available. Participant selection is presented in Figure 1. MCI was diagnosed in patients presenting with subjective and objective memory loss, without loss of function. This was consistent with Petersen’s criteria, where patients present with subjective memory complaints, objective abnormal memory function but preservation of activities of daily living and have no evidence of dementia [25]. Dementia was diagnosed using the Diagnostic and Statistical Manual of Mental Disorders (4th-edition) [26]. Mood was screened using the geriatric depression scale-short form with scores ≥5 assessed clinically for depression [27]. All participants were English literate. Those with MCI, predominantly amnestic type (aMCI), and patients with Alzheimer’s disease (AD), vascular dementia (VaD), frontotemporal dementia (FTD), Lewy body dementia (LBD) and Parkinson’s disease dementia (PDD), meeting established clinical criteria, with and without a history of comorbid depression, were included. Patients without cognitive impairment and with depression as the primary symptom were excluded. Persons attending with patients without memory loss (mainly caregivers) were recruited by convenience sampling as normal controls (*n* = 875). 

### 2.3. Outcome Measures

The SMMSE is a standardised form of the MMSE developed to improve inter-rater reliability and reduced administration time by using explicit administration and scoring guidelines [2,3]. Scored out of 30 points, a score of 25/30 or more suggests that the individual may have normal cognition. Below this, scores can indicate mild (21–24 points), moderate (10–20 points) and severe cognitive impairment (≤9 points), though a cut-off of <24/30 optimises sensitivity [2,3]. It covers several cognitive domains including orientation, registration, delayed (verbal) recall, attention (concentration and calculation), language (including writing, reading and naming), command following, and visuospatial (construction) subtests [2,3]. The Q*mci* screen is a more recently developed short CSI, designed to separate MCI from mild dementia [8]. Scored from 100 points it incorporates six subtests across five cognitive domains including orientation, working memory, sematic memory (verbal fluency-categories), visuo-spatial (clock drawing) and two tests of episodic memory: Delayed recall and logical memory (immediate verbal recall of short a story) [28]. It has an optimal cut-off score for cognitive impairment of <62/100 [13,29]. It has been validated against the MoCA and different neuropsychological tests and is published in multiple languages [30,31,32,33,34]. While data on total scores were available for both CSIs, data on subtests were only available for the Q*mci* screen as these were not collected as part of the DARAD trial [24]. 

### 2.4. Analysis

Data were analysed using SPSS 24.0. Data from the three data sets (original Q*mci* screen validation database, GAT and DARAD datasets) were pooled and analysed using simple descriptive statistics. Data were non-normally distributed and were analysed with non-parametric tests. The Mann–Whitney U test was used to compare distributions between variables. Receiver operating characteristic (ROC) curve analysis was used to determine diagnostic accuracy from the area under the curve (AUC). AUC scores range from 0.5–1.0; 0.5 equates to chance alone and 1.0 perfect predictive accuracy. Scores from 0.50–0.59 indicate no or very poor accuracy, 0.60–0.69 poor, 0.70–0.79 fair, 0.80–0.89 good and 0.90–1.0 excellent to perfect accuracy [35]. All AUC values are presented with 95% confidence intervals (CI) and where specified were compared using the DeLong method [36]. Optimal cut-off points were calculated using Youden’s Index.

## 3. Results

In all, 3020 patients were available for analysis. A further 875 normal controls were included. The majority of participants had dementia (*n* = 2160) of which AD was the most common subtype (*n* = 1483), followed by mixed (AD-VaD) (*n* = 400) and VaD (*n* = 130). The median age of patients presenting with cognitive symptoms (MCI/dementia) was 77 years, interquartile range (IQR) ±9 compared to a median age of 69 (±14) years for normal controls, *p* < 0.001. In all, 51% of patients were male compared to 43% of controls, *p* < 0.001. Patients had completed a median of 12 (±5) years in education, similar to controls (13 ± 4), albeit statistically significantly lower, *p* < 0.001. The median SMMSE scores were 23/30 (±8) for dementia, 28/30 (±4) for MCI and 29/30 (±2) for controls. Median Q*mci* screen scores were 38/100 (±26) for dementia, 56/100 (±20) for MCI and 74/100 (±15) for controls. Differences in gender were seen between diagnosis with the percentage of males ranging from as high as 73% in those with VaD to as low as 41% in those with dementia and comorbid depression. Characteristics of participants including patients and controls are presented in Table 1.

**Figure 2:**
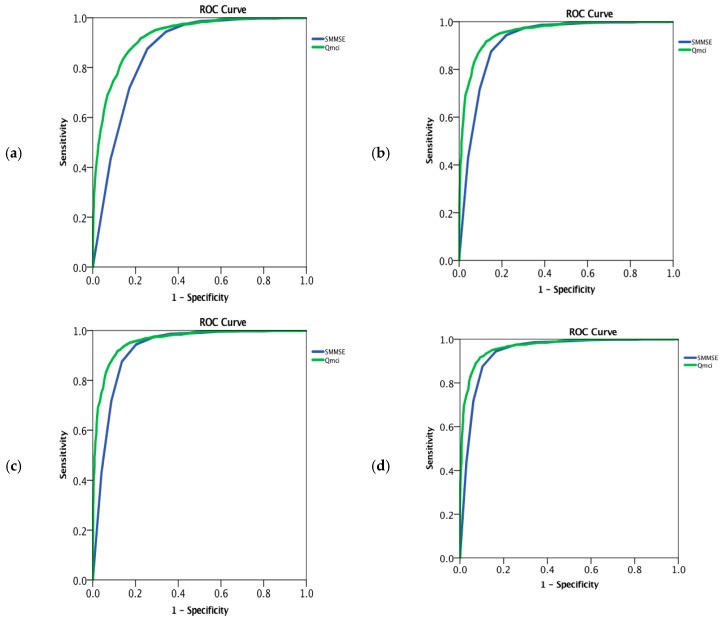

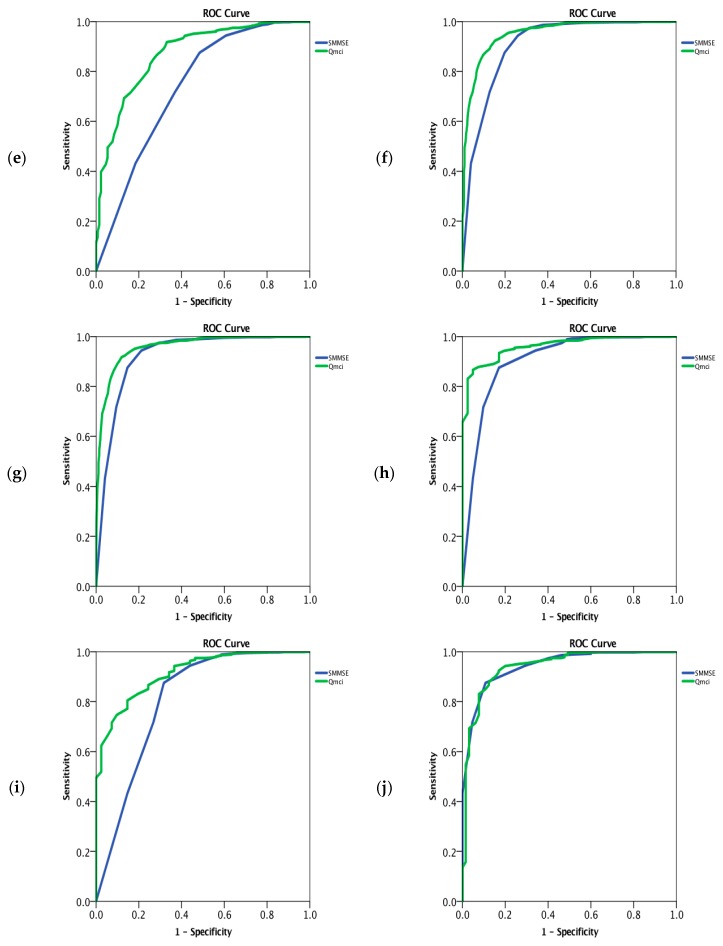

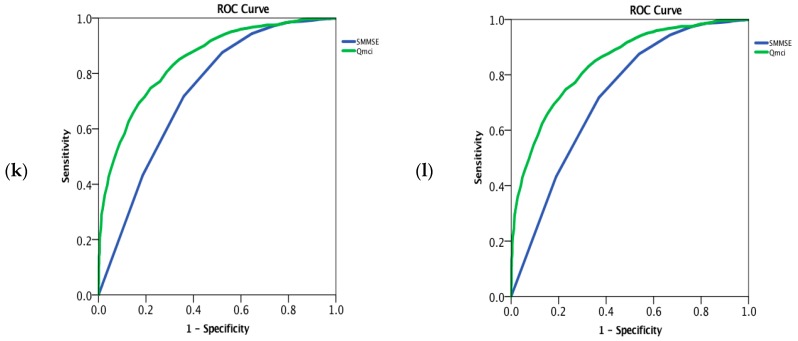
Receiver operating characteristic curves (**a**–**l**) comparing the accuracy of the Quick Mild Cognitive Impairment (Q*mci*) Screen and the Standardised Mini-Mental State Examination in differentiating normal controls (*n* = 875) from those with mild cognitive impairment (MCI), different dementia subtypes and comorbid depression for participants included within the Q*mci* screen validation, Doxycycline And Rifampicin for Alzheimer’s Disease study and Geriatric Assessment Tool databases. (**a**). All patients (*n* = 3020); (**b**). All patients with dementia (*n* = 2160); (**c**). Dementia excluding depression (*n* = 1879); (**d**). Alzheimer’s dementia (*n* = 1483); (**e**). Vascular dementia (*n* = 130); (**f**). Mixed dementia (*n* = 400); (**g**). Alzheimer’s, vascular, mixed dementia (*n* = 2013); (**h**). Frontotemporal dementia (*n* = 41) (**i**). Parkinson’s disease dementia (*n* = 41); (**j**). Lewy body dementia (*n* = 65); (**k**). All MCI (*n* = 860); (**l**) MCI excluding depression (*n* = 766).

Both instruments accurately differentiated cognitive impairment (MCI or dementia) from normal, although the Q*mci* screen was statistically more accurate than the SMMSE (AUC of 0.93 versus 0.87, respectively, *p* < 0.001). The SMMSE, at a cut-off of <24/30, had a sensitivity of 42%, specificity of 99% with a positive predictive value of 99% and negative predictive value of 33%. The Q*mci* screen had a sensitivity of 83% and specificity of 87% with a positive predictive value of 96% and negative predictive value of 60% at its published optimal cut-off score (<62/100). Using Youden’s Index, the optimal cut-off scores for the SMMSE was 28/30, which gave a sensitivity of 75% and specificity of 88%. The optimal cut-off for the Q*mci* screen was <62. The SMMSE most accurately differentiated AD (AUC 0.94, 95% CI: 0.93–0.95) and LBD (AUC 0.94, 95% CI: 0.92–0.97) and least accurately identified MCI (AUC 0.73, 95% CI: 0.71–0.75), VaD (AUC 0.74, 95% CI: 0.68–0.79) and PDD (AUC 0.81, 95% CI: 0.72–0.90). The Q*mci* screen had statistically greater accuracy in distinguishing all dementia subtypes except LBD (*p* = 0.91). The Q*mci* screen was more accurate than the SMMSE in separating PDD and FTD from controls, albeit sample sizes were small. As expected, the Q*mci* screen had the greatest diagnostic accuracy for identifying MCI (AUC 0.85, 95% CI: 0.83-0.87) from normal controls, which was statistically significantly greater than the SMMSE (AUC 0.73, 95% CI: 0.71–0.75), *p* < 0.001. ROC curves demonstrating the accuracy of both instruments in each type of dementia, in MCI and in those with and without depression are presented in Figure 2a–l.

The median subtest scores and AUC scores derived from ROC curves according to diagnosis are presented in Table 2 and Table 3, respectively.

**Figure 3:**
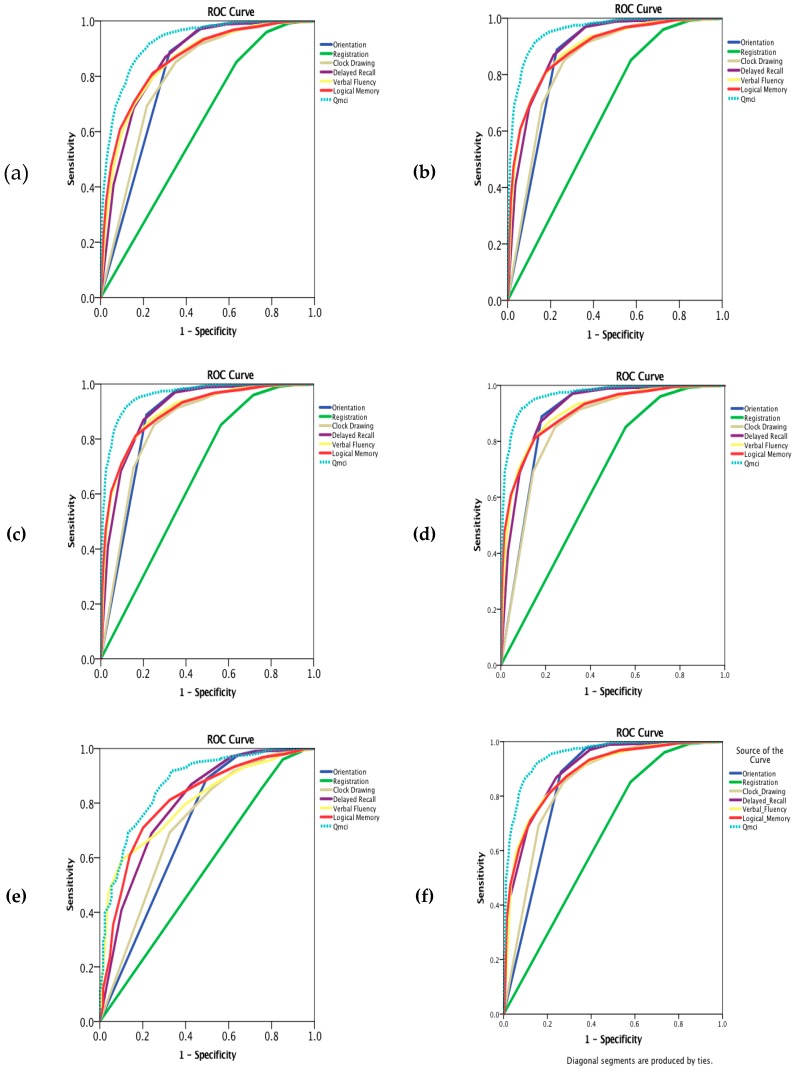

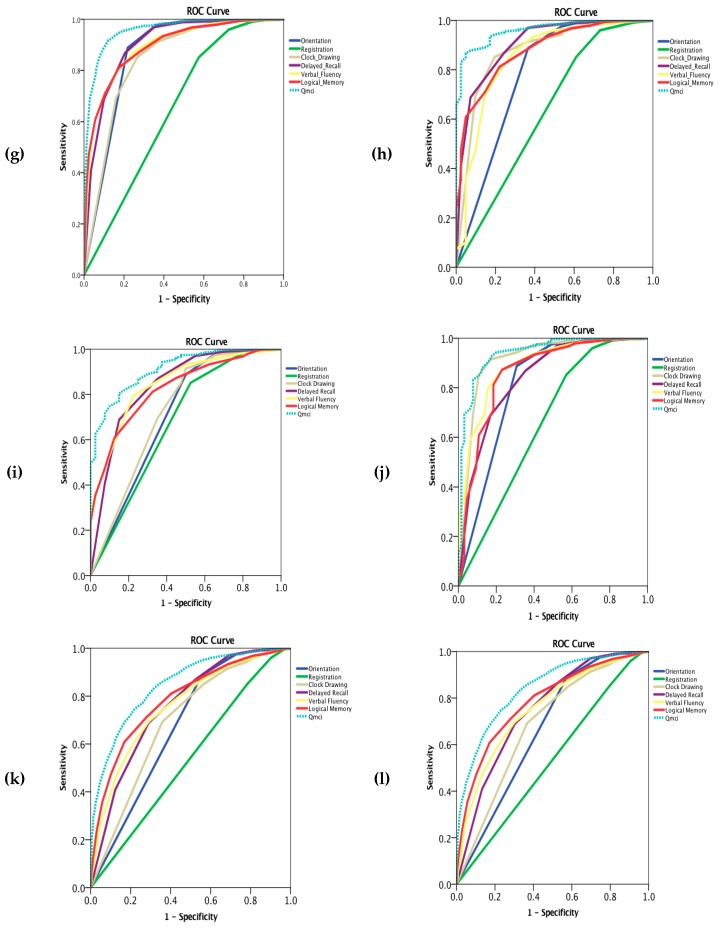
Receiver operating characteristic curves (**a**–**l**) comparing the accuracy of the Quick Mild Cognitive Impairment (Q*mci*) subtests in differentiating normal controls (*n* = 875) from those with mild cognitive impairment (MCI), different dementia subtypes and comorbid depression for participants included within the Q*mci* screen validation, Doxycycline and Rifampicin for Alzheimer’s Disease study and Geriatric Assessment Tool databases. (**a**). All patients (*n* = 3020); (**b**). All patients with dementia (*n* = 2160); (**c**). Dementia excluding depression (*n* = 1879); (**d**). Alzheimer’s dementia (*n* = 1483); (**e**). Vascular dementia (*n* = 130; (**f**). Mixed dementia (*n* = 400); (**g**). Alzheimer’s, vascular, mixed dementia (*n* = 2013); (**h**). Frontotemporal dementia (*n* = 41); (**i**). Parkinson’s disease dementia (*n* = 41); (**j**). Lewy body dementia (*n* = 65); (**k**). All MCI (*n* = 860); (**l**). MCI excluding depression (*n* = 766).

ROC curves comparing the subtests of the Q*mci* screen are presented in Figure 3a–l. The highest median score for the clock drawing subtest was found in those with VaD (14/15), the lowest was in LBD (7/15). The logical memory subtest was the most accurate of the Q*mci* screen subtests for most dementia subtypes and MCI (AUC 0.80, 95% CI: 0.77–0.81). Orientation was accurate for AD (AUC 0.88, 95% CI:) but had particularly low accuracy in VaD (AUC 0.71, 95% CI: 0.66–0.71), FTD (AUC 0.78, 95% CI: 0.69–0.87), PDD (AUC 0.71, 95% CI: 0.61–0.81) and MCI (AUC 0.68, 95% CI: 0.65–0.70). Clock drawing had the highest accuracy for identifying PDD (AUC 0.92, 95% CI: 0.87–0.96). Word registration had the lowest accuracy for all dementia subtypes and MCI.

Those diagnosed with dementia and co-morbid depression were younger (*z* = −5.9, *p* < 0.001) and more likely to be female (*X*^2^ = 11.4, *p* < 0.001) than those without comorbid depression. There was no statistically significant difference in the number of years in education (*z* = −1.3, *p* = 0.21). Those diagnosed with dementia (all subtypes excluding MCI) with co-morbid depression (*n* = 281) had statistically significantly higher median Q*mci* screen and SMMSE scores than those without (*n* = 1879) depression: Median Q*mci* screen scores of 44 versus 37, respectively (z = −4.771, *p* < 0.001) and median SMMSE scores of 25 versus 23, respectively (z = −5.627, *p* < 0.001). Contrasting this, comparison of median scores for MCI with and without depression showed that scores were significantly lower for those with co-morbid depression for both the Q*mci* screen, (52 and 57 respectively, z = −2.927, *p* = 0.003) and SMMSE (26 and 28 respectively, z = −3.302, *p* = 0.001). Co-morbid depression lowered the diagnostic accuracy of both instruments for dementia but improved the accuracy in those with MCI. All Q*mci* screen subtest scores were less accurate for MCI among those patients with co-morbid depression.

## 4. Discussion

This study compares the diagnostic accuracy of the SMMSE and Q*mci* screen in different dementia subtypes using a large sample of patients pooled from three different datasets using AUC values as a global measure of diagnostic accuracy. The results show that the SMMSE and Q*mci* screen are both accurate CSIs when used to identify dementia in patients presenting with cognitive symptoms to geriatric and memory clinics compared with normal controls. Overall, the Q*mci* screen had high sensitivity and specificity in separating normal controls from those with cognitive impairment (MCI or dementia with or with co-morbid depression). The SMMSE had poor sensitivity, albeit excellent specificity at its widely-used cut-off. These results would be expected as the Q*mci* screen contains more challenging tests of episodic memory [28], which are better able to differentiate MCI from mild dementia [8,29]. The SMMSE is overly weighted towards tests of orientation (one-third of its points), which is best able to identify established dementia [28]. Further, while a cut-off of <24/30 is widely applied for the SMMSE, recent studies suggest that higher cut-offs between 26 [37] and 29 [13], closer to that found here, are more accurate and produce a better balance between sensitivity and specificity. As with its’ original validation study [8,28], this analysis confirms the Q*mci* screen is more accurate overall and that its logical memory subtest is its most accurate for separating MCI from normal controls. It also had high levels of accuracy for most dementia subtypes and patients with and without co-morbid depression. The SMMSE, while it had good to excellent accuracy in differentiating most dementia subtypes from normal controls, was less accurate in identifying MCI [8], and had only fair accuracy in identifying VaD (AUC of 0.74) from controls, supporting previous studies in these conditions, where an alternative instrument is suggested [17]. Similarly, while the SMMSE’s accuracy in detecting PDD from normal controls was good (AUC 0.81), it performed relatively poorly compared to the Q*mci* screen, supporting evidence that it is less suitable due to both floor and ceiling effects in those with movement disorders [18,38]. The study also examined the subtests of the Q*mci* screen and their differential accuracy in separating those with MCI and dementia from normal controls. As was found in the initial validation, logical memory was most accurate in identifying MCI [28]. Clock drawing was most accurate in detecting LBD but the accuracy for PDD was relatively lower. Clock drawing is often grossly abnormal in LBD, particularly for copying rather than drawing clocks [39]. Differences between LBD and PDD were unexpected and it is likely that small numbers influenced the results. Orientation was only accurate for AD and mixed dementia having poor accuracy for other dementia subtypes and MCI.

In this study, co-morbid depression had a significant impact on CSI scores; those with dementia and depression scored significantly higher on both the SMMSE and Q*mci* screen than those without. This was unexpected as other studies [22] suggest that impaired attention and other cognitive deficits associated with low mood negatively impact on scores. Nevertheless, co-morbid depression did lower the diagnostic accuracy of both instruments for differentiating dementia from normal controls. The opposite effect was seen for MCI; those with co-morbid depression scored less well on both CSIs, which had higher diagnostic accuracy for these patients. This suggests that comorbid depression may influence CSIs in different ways depending on the diagnostic stage of cognitive impairment. Depression may also have a greater clinical effect at earlier stages of disease progression with evidence that depressive symptoms increase the risk for converting from MCI to dementia, particularly amnestic type to AD [21,40].

The strength of this study is derived from its large sample size and careful pooling of data derived from similar data sets collected by the same principal investigator. This study also has limitations. Pooling data from discrete, albeit related, datasets with different populations may have created bias. Some data on gender were missing and subtest data for the SMMSE were not available across all three datasets, which limited the analysis. The number of patients with atypical or less common dementia subtypes was small and may be unrepresentative of the true performance of the instruments in that subtype, leading to bias. The data collection began in 1999 when the awareness of LBD was low, potentially resulting in misclassification bias. Likewise, only patients with AD or those with AD, VaD and mixed dementia were included in the DARAD and the Q*mci* validation databases respectively. In addition, the prevalence of cognitive impairment (MCI/dementia) was high among those attending these geriatric and memory clinics potentially leading to spectrum bias. Further, this study was a retrospective review of patients with no detailed information available regarding the type of depressive symptoms. Similarly, it was not possible to assess MCI subtypes in this study, though the majority of patients in the GAT database reported amnestic type symptoms, suggesting that most had aMCI. Finally, the statistical analysis was limited to using AUC scores as a global measure of diagnostic accuracy and further research is now planned to explore the psychometric properties of these instruments in different dementia subtypes and to identify the optimal cut-off scores.

In summary, this study reaffirms that both the SMMSE and Q*mci* screen are useful in separating patients with dementia from normal controls. It confirms the superior accuracy of the Q*mci* screen in MCI. It also shows that different short screens have significantly different accuracy in different dementia subtypes, suggesting that pre-screen/pre-test suspicion of a possible diagnosis (MCI or specific dementia subtype) based upon history and examination should direct the choice of instrument to be used when performing cognitive screening. This is important as time is limited in clinical practice and short CSIs can only include a limited number of domains, resulting in a time-accuracy trade-off supporting the need for careful selection a priori [41]. The results also suggest that a history of depression may affect the accuracy of cognitive screens, particularly in those with MCI in which it lowers median scores but increased the diagnostic accuracy of CSIs. The opposite effect was seen in dementia. This shows the importance of asking about depression when undertaking cognitive screening [20] and that the effect of comorbid depression on cognitive screening scores merits further investigation. Understanding the optimal cut-offs for these instruments and other short CSIs is also important, so that not only the most appropriate instrument is used in the right setting but also the correct cut-off score is applied. To date, no condition-specific cut-off scores for the SMMSE or Q*mci* screen are available, highlighting that this is an area requiring more research. Further study is also required to confirm these findings and compare with sensitive and specific instruments such as the MoCA and the Mini-Addenbrooke’s Cognitive Examination [42].

## Figures and Tables

**Table 1:** Characteristics of participants from the Quick Mild Cognitive Impairment (Q*mci*) Screen validation, Doxycycline And Rifampicin for Alzheimer’s Disease study and Geriatric Assessment Tool databases, divided according to diagnosis: Depression, controls, mild cognitive impairment (MCI) and dementia including their Standardised Mini-Mental State Examination (SMMSE) and Q*mci* screen scores; comparison of SMMSE and Q*mci* screen score accuracy in differentiating each diagnosis from controls.

Diagnosis	*N* = X	Age (Median & IQR)	Education (Median & IQR)	Gender (% Male)	SMMSE (Median & IQR)	Q*mci* (Median & IQR)	SMMSE AUC NC v CI (95% Confidence Intervals)	Q*mci* AUC NC v CI (95% Confidence Intervals)	*p* = x
Total (All including co-morbid depression & MCI)	3020	77 (81 − 72 = 9)	12 (14 − 9 = 5)	1537/2997 * (51%)	25 (28 − 20 = 8)	43 (56 − 29 = 27)	0.87 (0.85–0.88)	0.93 (0.92–0.94)	*z* = 11.6 *p* < 0.001
Dementia (All including co-morbid depression)	2160	77 (82 − 73 = 9)	12 (14 − 9 = 5)	1087/2145 * (51%)	23 (26 − 18 = 8)	38 (50 − 24 = 26)	0.92 (0.91–0.93)	0.96 (0.95–0.96)	*z* = −8.6 *p* < 0.001
Dementia (All excluding co-morbid depression)	1879	78 (82 − 74 = 8)	11 (14 − 9 = 5)	971/1864 * (52%)	23 (26 − 18 = 8)	37 (49 − 24 = 25)	0.92 (0.91–0.93)	0.96 (0.95–0.97)	*z* = −8.3 *p* < 0.001
AD	1483	78 (83 − 74 = 9)	12 (14 − 9 = 5)	651/1475 * (44%)	23 (25.5 − 18 = 7.5)	37 (48 − 23 = 25)	0.94 (0.93–0.95)	0.97 (0.96–0.97)	*z* = −5.9 *p* < 0.001
VaD	130	74 (79 − 69 = 10)	12 (14 − 10 = 4)	95 (73%)	27 (29 − 25 = 4)	53 (64 − 40 = 24)	0.74 (0.68–0.79)	0.87 (0.84–0.91)	*z* = −6.3 *p* < 0.001
Mixed (AD/VaD)	400	77 (81 − 74 = 7)	11 (13 − 9 = 4)	256/393 * (65%)	22 (27 − 19 = 8)	36 (51 − 23 = 28)	0.91 (0.89–0.93)	0.95 (0.94–0.96)	*z* = −5.7 *p* < 0.001
AD, VaD and Mixed	2013	78 (82 − 74 = 4)	12 (14 − 9 = 5)	1002/1998 * (50%)	23 (26 − 18 = 8)	38 (50 − 24 = 26)	0.92 (0.91–0.93)	0.96 (0.95–0.97)	*z* = 8.4 *p* < 0.001
FTD	41	69 (71 − 62 = 9)	12 (14 − 10 = 4)	25/41 (61%)	23 (27 − 19 = 8)	42 (52 − 28 = 24)	0.90 (0.85–0.96)	0.96 (0.94–0.98)	*z* = −2.5 *p* = 0.01
PDD	41	75 (77 − 71 = 6)	12 (12 − 9.5 = 2.5)	28/41 (68%)	26 (29 − 21 = 8)	46 (61 − 32 = 29)	0.81 (0.72–0.90)	0.92 (0.88–0.95)	*z* = −3.1 *p* = 0.002
LBD	65	78 (82 − 73 = 9)	10 (14 − 8 = 6)	32/65 (49%)	24 (27 − 18 = 9)	37 (54 − 24 = 30)	0.94 (0.92–0.97)	0.94 (0.91–0.97)	*z* = 0.11 *p* = 0.91
MCI	860	75 (80 − 70 = 10)	12 (14 − 10 = 4)	450/852 * (53%)	28 (29 − 25 = 4)	56 (66 − 46 = 20)	0.73 (0.71–0.75)	0.85 (0.83–0.87)	*z* = −10.8 *p* < 0.001
Co-morbid depression	281	75 (81 − 70 = 11)	12 (14 − 10 = 4)	116/281 (41%)	25 (27 − 21 = 6)	44 (56 − 30 = 26)	0.88 (0.86–0.91)	0.93 (0.91–0.95)	*z* = −4.2 *p* < 0.001
MCI with co-morbid depression	94	73 (75 − 68 = 7)	12 (14 − 9 = 5)	50/92 * (54%)	26 (29 − 23 = 6)	52 (62 − 40 = 22)	0.79 (0.73–0.85)	0.90 (0.86–0.93)	*z* = −4.4 *p* < 0.001
MCI without co-morbid depression	766	76 (80 − 70 = 10)	12 (14 − 10 = 4)	400/760 * (53%)	28 (29 − 26 = 3)	57 (66 − 46 = 20)	0.72 (0.70–0.75)	0.84 (0.82–0.86)	*z* = −10.4 *p* < 0.001
Controls	875	70 (76 − 62 = 14)	13 (16 − 12 = 4)	372/875 (43%)	29 (30 − 28 = 2)	74 (81 − 66 = 15)	NA	NA	NA

AUC = area under the curve, AD = Alzheimer’s disease, CI = cognitive impairment, FTD = frontotemporal dementia, IQR = interquartile range, LBD = Lewy body dementia, PDD = Parkinson’s dementia, VaD = vascular dementia; NA = Not applicable; * Missing data.

**Table 2:** Median scores for the Quick Mild Cognitive Impairment (Q*mci*) Screen subtests divided according to diagnosis: Depression, mild cognitive impairment (MCI) and dementia.

Diagnosis	*N* = X	Orientation (Median and IQR)	Registration (Median and IQR)	Clock Drawing (Median and IQR)	Delayed Recall (Median and IQR)	Verbal Fluency (Median and IQR)	Logical Memory (Median and IQR)	Q*mci* Total (Median and IQR)
Total (All)	3020	8 (10 − 6 = 4)	5 (5 − 4 = 1)	12 (14 − 5 = 9)	4 (12 − 0 = 12)	5 (7 − 3 = 4)	8 (12 − 4 = 8)	43 (56 − 29 = 27)
Dementia (All including co-morbid depression)	2160	7 (9 − 5 = 4)	5 (5 − 3 = 2)	11 (14 − 3 = 11)	4 (8 − 0 = 8)	5 (7 − 3 = 4)	8 (12 − 4 = 8)	38 (50 − 24 = 26)
Dementia (All excluding co-morbid depression)	1879	7 (9 − 5 = 4)	5 (5 − 3 = 2)	11 (13 − 3 = 10)	0 (8 − 0 = 8)	4 (6 − 3 = 3)	8 (12 − 4 = 8)	37 (49 − 24 = 25)
AD	1483	7 (9 − 5 = 4)	5 (5 − 3 = 2)	11 (13 − 3 = 10)	0 (8 − 0 = 8)	4 (6 − 3 = 3)	8 (12 − 4 = 8)	37 (48 − 23 = 25)
VaD	130	9 (10 − 7 = 3)	5 (5 − 4 = 1)	14 (15 − 11 = 4)	8 (12 − 0 = 12)	7 (9 − 4 = 5)	10 (14 − 8 = 6)	53 (64 − 40 = 24)
Mixed (AD/VaD)	400	7 (10 − 5 = 5)	5 (5 − 3 = 2)	10 (14 − 2 = 12)	0 (8 − 0 = 8)	5 (7 − 3 = 4)	8 (12 − 4 = 8)	35.5 (51 − 23 = 28)
AD, VaD and Mixed	2013	7 (9 − 5 = 4)	5 (5 − 3 = 2)	11 (14 − 3 = 11)	0 (8 − 0 = 8)	5 (7 − 3 = 4)	8 (12 − 4 = 8)	38 (50 − 24 = 26)
FTD	41	9 (10 − 6 = 4)	5 (5 − 3 = 2)	12 (13 − 5 = 8)	4 (8 − 0 = 8)	4 (7 − 3 = 4)	8 (12 − 4 = 8)	43 (55 − 30 = 25)
PDD	41	9.5 (10 − 7 = 3)	5 (5 − 4 = 1)	12.5 (15 − 7 = 8)	8 (12 − 0 = 12)	5.5 (7 − 4 = 3)	10 (14 − 8 = 6)	46 (61 − 32 = 29)
LBD	65	8 (10 − 6 = 4)	5 (5 − 3 = 2)	7 (12 − 1 = 11)	8 (12 − 0 = 12)	4 (6 − 3 = 3)	8 (10 − 4 = 6)	37 (54 − 24 = 30)
MCI	860	10 (10 − 8 = 2)	5 (5 − 5 = 0)	14 (15 − 12 = 3)	12 (16 − 4 = 12)	7 (9 − 5 = 4)	12 (16 − 8 = 8)	56 (66 − 46 = 20)
Co-morbid depression	281	8 (10 − 6 = 4)	5 (5 − 4 = 1)	11 (14 − 4 = 10)	4 (12 − 0 = 12)	6 (8 − 3 = 5)	10 (14 − 4 = 10)	44 (56 − 30 = 26)
MCI with co-morbid depression	94	9 (10 − 7 = 3)	5 (5 − 4 = 1)	13 (15 − 10 = 5)	8 (12 − 4 = 8)	8 (8 − 4 = 4)	12 (14 − 8 = 6)	51.5 (62 − 40 = 22)
MCI without co-morbid depression	766	10 (10 − 8 = 2)	5 (5 − 5 = 0)	14 (15 − 12 = 3)	12 (16 − 4 = 12)	7 (9 − 5 = 4)	12 (16 − 8 = 8)	57 (66 − 46 = 20)
Controls	875	10 (10 − 10 = 0)	5 (5 − 5 = 0)	15 (15 − 14 = 1)	16 (20 − 12 = 8)	10 (13 − 8 = 5)	18 (22 − 14 = 8)	74 (81 − 66 = 15)

AD = Alzheimer’s disease, FTD = frontotemporal dementia, LBD = Lewy body dementia, PDD = Parkinson’s dementia, VaD = vascular dementia (IQR = interquartile range).

**Table 3:** Comparison of the accuracy of the Quick Mild Cognitive Impairment (Q*mci*) Screen subtests in differentiating each diagnosis from controls (*n* = 875): Depression, mild cognitive impairment (MCI) and dementia.

Diagnosis	*N* = X	Orientation AUC (95% CI)	Registration AUC (95% CI)	Clock Drawing AUC (95% CI)	Delayed Recall AUC (95% CI)	Verbal Fluency AUC (95% CI)	Logical Memory AUC (95% CI)	Q*mci* Total AUC (95% CI)
Total (All)	3020	0.81 (0.79–0.82)	0.62 (0.60–0.64)	0.80 (0.79–0.82)	0.86 (0.85–0.87)	0.85 (0.84–0.87)	0.87 (0.85–0.88)	0.93 (0.92–0.94)
Dementia (All including co-morbid depression)	2160	0.86 (0.85–0.87)	0.65 (0.63–0.67)	0.85 (0.83–0.86)	0.90 (0.89–0.91)	0.89 (0.88–0.90)	0.89 (0.88–0.91)	0.96 (0.95–0.96)
Dementia (All excluding co-morbid depression)	1879	0.87 (0.85–0.88)	0.66 (0.64–0.68)	0.85 (0.84–0.87)	0.90 (0.89–0.92)	0.90 (0.88–0.91)	0.90 (0.89–0.91)	0.96 (0.95–0.97)
AD	1483	0.88 (0.87–0.90)	0.66 (0.64–0.68)	0.85 (0.84–0.87)	0.91 (0.90–0.93)	0.91 (0.89–0.92)	0.91 (0.89–0.92)	0.97 (0.96–0.97)
VaD	130	0.71 (0.66–0.77)	0.56 (0.50–0.61)	0.72 (0.67–0.77)	0.80 (0.75–0.84)	0.80 (0.77–0.84)	0.81 (0.77–0.85)	0.87 (0.84–0.91)
Mixed (AD/VaD)	400	0.84 (0.82–0.87)	0.65 (0.61–0.68)	0.85 (0.82–0.87)	0.90 (0.88–0.92)	0.89 (0.87–0.91)	0.89 (0.87–0.91)	0.95 (0.94–0.96)
AD, VaD & Mixed	2013	0.86 (0.85–0.88)	0.65 (0.63–0.67)	0.84 (0.83–0.86)	0.90 (0.89–0.91)	0.90 (0.88–0.91)	0.90 (0.88–0.91)	0.96 (0.95–0.97)
FTD	41	0.78 (0.69–0.87)	0.63 (0.53–0.73)	0.88 (0.81–0.94)	0.90 (0.87–0.95)	0.85 (0.78–0.92)	0.88 (0.83–0.93)	0.96 (0.94–0.98)
PDD	41	0.71 (0.61–0.81)	0.68 (0.58–0.77)	0.73 (0.63–0.82)	0.84 (0.77–0.91)	0.85 (0.79–0.91)	0.83 (0.77–0.88)	0.92 (0.88–0.95)
LBD	65	0.82 (0.74–0.88)	0.66 (0.58–0.74)	0.92 (0.87–0.96)	0.84 (0.79–0.90)	0.89 (0.85–0.93)	0.86 (0.81–0.92)	0.94 (0.91–0.97)
MCI	860	0.68 (0.65–0.70)	0.54 (0.51–0.57)	0.69 (0.67–0.72)	0.76 (0.74–0.78)	0.76 (0.74–0.78)	0.80 (0.77–0.81)	0.85 (0.83–0.87)
Co-morbid depression	281	0.80 (0.77–0.84)	0.62 (0.57–0.66)	0.81 (0.78–0.84)	0.86 (0.84–0.89)	0.84 (0.82–0.87)	0.84 (0.82–0.87)	0.93 (0.91–0.95)
MCI with co-morbid depression	94	0.75 (0.69–0.82)	0.59 (0.52–0.65)	0.75 (0.70–0.81)	0.82 (0.78–0.87)	0.80 (0.76–0.85)	0.80 (0.75–0.84)	0.90 (0.86–0.93)
MCI without co-morbid depression	766	0.67 (0.64–0.69)	0.53 (0.50–0.56)	0.69 (0.66–0.71)	0.75 (0.73–0.77)	0.76 (0.73–0.78)	0.80 (0.76–0.81)	0.84 (0.82–0.86)

AD = Alzheimer’s disease, FTD = frontotemporal dementia, LBD = Lewy body dementia, PDD = Parkinson’s dementia, VaD = vascular dementia; AUC = area under the curve; CI = confidence intervals.
